# Characterization of Nutritional and Flavor Profiles of Aromatic Black Rice, India

**DOI:** 10.1155/ijfo/5759996

**Published:** 2026-07-08

**Authors:** Babli Waribam, Raju Sasikumar, Selva Kumar T., Phani Kumar Garlapati, Sandeep Janghu, S. Chakkaravarthi, Paul Mansingh, Vidisha Tomer, Amit K. Jaiswal

**Affiliations:** ^1^ Department of Agribusiness Management and Food Technology, North-Eastern Hill University, Chasingre, Tura, Meghalaya, India, nehu.ac.in; ^2^ Vel Tech Rangarajan Dr. Sagunthala R&D Institute of Science and Technology, Chennai, India, veltech.edu.in; ^3^ Defence Institute of Biodefence Technologies (DIBT-DRDO), Mysore, Karnataka, India, drdo.gov.in; ^4^ Department of Food Technology, Rajiv Gandhi University, Doimukh, Arunachal Pradesh, India, rgu.ac.in; ^5^ Department of Interdisplinary Sciences, National Institute of Food Technology Entrepreneurship and Management, Sonipat, Haryana, India, niftem.ac.in; ^6^ Department of Agricultural Extension and Economics, VIT School of Agricultural Innovations and Advanced Learning (VAIAL), Vellore Institute of Technology, Vellore, India, vit.ac.in; ^7^ Department of Horticulture and Food Science, VIT School of Agricultural Innovations and Advance Learning, Vellore Institute of Technology, Vellore, Tamil Nadu, India, vit.ac.in; ^8^ Centre for Sustainable Packaging and Bioproducts (CSPB), Technological University Dublin—City Campus, Dublin, Ireland; ^9^ School of Food Science and Environmental Health, Technological University Dublin—City Campus, Dublin, Ireland

**Keywords:** FAMEs, GC-MS/MS, ICP-MS, volatile compounds

## Abstract

*Chakhao* is an indigenous aromatic black rice variety from Manipur, India, valued for its distinctive flavor, high anthocyanin content, and associated health benefits. Despite its geographical indication status, limited information exists on how regional cultivation conditions influence its compositional attributes. This study characterizes and compares the nutritional composition, mineral content, volatile profile, and fatty acid methyl esters (FAMEs) composition of *Chakhao* sourced from five major cultivation regions in Manipur. Proximate analysis quantified moisture, protein, ash, fiber, fat, and carbohydrate content, whereas GC‐MS/MS and ICP‐MS were employed to profile volatile compounds, FAMEs, and mineral elements. Carbohydrates were the predominant macronutrient (73.88%–74.21%), with protein levels ranging from 10.19% to 10.89%. Potassium (238.14–348.42 mg/100 g) was the most abundant mineral, followed by magnesium (93.88–145.74 mg/100 g). Key volatile compounds such as hexanal, nonanal, butanal, and region‐specific variations in azulene and 1‐hexanol contributed to the characteristic aroma of *Chakhao*. Fatty acid profiling identified oleic acid (37.00%–44.04%) as the dominant monounsaturated fatty acid, linoleic acid (30.27%–35.84%) as the major polyunsaturated fatty acid, and palmitic acid (18.38%–20.51%) as the principal saturated fatty acid. This study provides the first integrative, region‐wise biochemical assessment of *Chakhao*, highlighting its nutritional richness and aroma complexity, and emphasizing the need for standardized cultivation and processing practices to ensure consistent quality and support its valorization in functional food applications.

## 1. Introduction

Rice (*Oryza sativa* L.) is a staple food for nearly half of the global population and is characterized by remarkable genetic, nutritional, and sensory diversity, with more than 110,000 documented varieties [1]. India, one of the largest rice‐producing countries, maintains over 70,000 germplasms [2], including several pigmented and aromatic landraces that are increasingly valued for their superior nutrient density and bioactive compound content. Among these, black rice has gained particular attention due to its high anthocyanin concentration, antioxidant activity, and distinctive sensory attributes.

In northeastern India, black rice is represented by *Chakhao*, an indigenous aromatic variety cultivated across the hilly and lowland regions of Manipur. *Chakhao* is culturally significant, nutritionally rich, and visually characterized by its deep purple‐to‐black pigmentation, attributed primarily to anthocyanins [3]. Its unique identity and geographical specificity were officially recognized with the granting of geographical indication (GI) status in 2019 [4]. The grain is widely regarded for its sticky texture, health‐promoting phytochemicals, and distinctive aroma, positioning it as a potential high‐value functional food with expanding national and international demand.

Although several studies have examined the general nutritional or phytochemical attributes of pigmented rice [5, 6], comprehensive investigations into the combined nutritional, volatile, and lipid characteristics of *Chakhao* are extremely limited. Most existing research focuses on single‐parameter evaluations or comparisons with other pigmented rice types, and few address how geographical variation within Manipur influences *Chakhao*′s biochemical composition, despite the region′s heterogeneity in soil type, altitude, and microclimate. Such factors are known to influence mineral uptake, fatty acid biosynthesis, and volatile formation in rice, yet systematic region‐wise characterization of *Chakhao* has not been reported.

Furthermore, the volatile aroma profile of *Chakhao*, distinct from typical aromatic rice that is dominated by 2‐acetyl‐1‐pyrroline (2‐AP), is insufficiently documented. Reported compounds such as hexanal, nonanal, azulene, and 1‐hexanol [7] suggest that *Chakhao* possesses a unique aromatic signature. However, no study has compared volatile profiles across multiple *Chakhao* growing districts, limiting understanding of how environmental or agronomic factors contribute to flavor differentiation, which is an essential aspect for GI products and quality standardization.

Similarly, black rice is known to contain functional lipids, particularly oleic, linoleic, and palmitic acids, which influence both nutritional value and oxidative stability [8, 9]. Yet, region‐specific fatty acid profiling of *Chakhao* remains unexplored, leaving a knowledge gap regarding variability in lipid composition and its implications for shelf life, sensory quality, and functional food development.

Given these gaps, there is a clear need for a multidimensional compositional study that integrates proximate analysis, mineral profiling, volatile compound identification, and fatty acid characterization across *Chakhao* samples grown in different agroecological regions of Manipur. Such an approach is crucial for establishing scientific baselines for quality control, understanding environmental influences on grain biochemistry, and supporting value‐chain development for this culturally significant GI‐protected crop.

Therefore, the present study is aimed at systematically characterizing the nutritional composition, mineral content, aroma profile, and fatty acid composition of *Chakhao* sourced from five major cultivation regions in Manipur. Utilizing advanced analytical tools such as GC‐MS/MS and inductively coupled plasma mass spectrometry (ICP‐MS), this work provides the first region‐comparative dataset on *Chakhao*′s biochemical attributes. The findings are expected to support improved standardization, enhance market positioning, and facilitate the incorporation of *Chakhao* into functional food and nutraceutical applications.

## 2. Materials and Methods

### 2.1. Chemicals and Reagents

All chemicals, solvents, and standards used in this study were of analytical grade or high‐performance liquid chromatography (HPLC) grade. Reagents were procured from Sigma‐Aldrich, Mumbai, India.

### 2.2. Sample Collection

**Figure 1:**
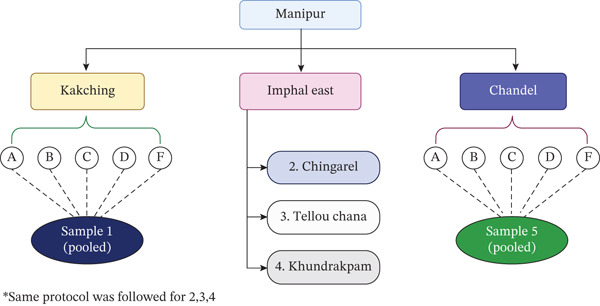
Sample collection methodology.

A purposive sampling method was employed to select *Chakhao* rice samples for this study [10]. Out of the 16 districts in Manipur, three districts with the highest production were selected. The specific geographical locations of the sample collection sites are depicted in Figure 1. Within each district, the headquarters served as the primary sampling region. Five locations with varying altitudes were randomly selected from the district headquarters to ensure representative sampling. Samples from five different regions were included in this study to observe how the nutritional composition of *Chakhao* differs from different cultivation regions. The coordinates of the sampling sites were as follows:

• 93°59 ^′^11.611 ^″^E & 24°30′52.328 ^″^N.
• 93°59 ^′^41.915 ^″^E & 24°51′23.397 ^″^N.
• 94°5 ^′^7.356 ^″^E & 24°52′18.931 ^″^N.
• 94°0 ^′^28.563 ^″^E & 24°19′46.01 ^″^N.
• 93°59 ^′^15.468 ^″^E & 24°53′15.649 ^″^N.

These five sampling sites were selected based on factors such as availability of the crop, elevation, and accessibility. *Chakhao*′s in these areas are naturally cultivated in rainfed environments in alluvial soil lands, with sowing through the monsoon and harvests in late October to November. Cultivators use traditional low‐input practices, depending on organic fertilizer and least modernization, which conserves the original characters of the crop. The altitudinal variation between locations influences conditions like temperature, rainfall, and soil moisture, thus leading to the variations in grain quality [11].

The collected rice samples from each location were pooled to ensure a comprehensive representation of the district′s *Chakhao* production. Standardized protocols were followed throughout the sampling process to maintain consistency and reliability in subsequent analyses.

### 2.3. Sample Preparation

Paddy samples were dehusked using a laboratory‐grade rice‐dehusking machine (Satake Rice Machine, Tokyo, Japan) to obtain brown rice. The dehusked grains were then milled into whole flour using a Philips HL7756/00 grinder, ensuring uniform particle size. The resulting flour was passed through a 60 BSS (250 *μ*m) sieve, and any coarse fractions were reground and sieved again until the desired particle size was achieved [12]. The processed samples were stored in airtight containers at room temperature until further analysis.

### 2.4. Proximate Analysis

The proximate composition, including moisture, protein, ash, fiber, and carbohydrate content, was determined following AOAC (2005) standard methods. All measurements were performed in triplicate.

#### 2.4.1. Determination of Moisture

Moisture content was determined using the AOAC 930.15 method [13]. A 5 g of the rice flour sample was weighed into a predried, tared porcelain dish and dried in a hot air oven (Model‐BTI‐HAO‐50, India) at 135°C for 2 h. The dish was then transferred to a desiccator to cool before reweighing. Moisture content was calculated using the equation:

Moisture%=W12−W×100W1−W,

where *W* is the weight of the empty dish (g), *W*1 is the weight of the dish with the sample before drying (g), and *W*2 is the weight of the dish with the sample after drying (g).

#### 2.4.2. Determination of Total Ash

Total ash content was determined using the AOAC International Method 900.02 [14]. A 2 g of each rice flour sample was weighed into a predried porcelain crucible. The sample was initially charred over a direct flame until visible fumes were no longer emitted. The crucible was then transferred to a muffle furnace (Milestone PYRO, Italy) set at 550°C–600°C and incinerated for 5 h until constant weight was obtained. After completion of incineration, the crucible was removed from the furnace and placed in a desiccator to cool down to room temperature before weighing. The total ash content was calculated using the equation:

Total ash content %=W2−W×100W1−W,

where *W*2 is the weight of the crucible with ash (g), *W* is the weight of the empty crucible (g), *W*1 is the weight of the crucible with the dried sample before incineration (g).

#### 2.4.3. Determination of Total Protein Content

The total protein content of *Chakhao* rice samples was determined using the Kjeldahl method following AOAC 2005 guidelines [15]. A 0.5 g of the sample was accurately weighed into a Kjeldahl digestion flask (Digestor DT 208, Denmark), followed by the addition of 1 g of a catalyst mixture (10:1 ratio of potassium sulfate (K_2_SO_4_) to copper sulfate (CuSO_4_) and 25 mL of concentrated sulfuric acid (H_2_SO_4_). The digestion was carried out in a heating mantle inside a fume chamber until a clear ammonium sulfate solution was obtained, indicating the complete breakdown of organic matter. The digested sample was allowed to cool to room temperature before proceeding to distillation.

The digested solution was transferred to a distillation unit (Kjeltech 800 nitrogen analyzer, Denmark), and sodium hydroxide (NaOH) was added to convert ammonium sulfate into ammonia gas. The released ammonia was distilled into a boric acid solution in a receiving flask. The borate‐ammonia complex formed in the receiving flask was titrated against standardized 0.1 N H_2_SO_4_ using methyl red as an indicator until the endpoint was reached. A blank titration was performed under identical conditions to account for any reagent contamination.

The nitrogen content was determined and converted to total protein using the standard nitrogen‐to‐protein conversion factor:

Protein%=N×6.25,

where **N** represents the nitrogen content (g) calculated from titration data, and 6.25 is the nitrogen‐to‐protein conversion factor for rice.

#### 2.4.4. Determination of Crude Fiber

Crude fiber content in the dried *Chakhao* rice samples was determined using acid digestion followed by alkali treatment, as described by Sadasivam and Manickam [16]. The analysis was conducted using the Fibra Plus analyzer (FES 04 AS DLS TS, India) to ensure precision. A 2 g of the sample was weighed and subjected to sequential digestion. First, the sample was boiled in 1.25% H_2_SO_4_ solution for 30 min, maintaining a consistent volume. The residue was then collected and boiled in 1.25% NaOH solution for another 30 min. The digested mixture was allowed to cool to room temperature, after which it was filtered through a glass crucible containing celite powder. The residue was thoroughly washed with distilled water until the filtrate became clear. The filtered residue was transferred to a hot air oven and dried at 130°C for 2 h. After drying, the sample was cooled in a desiccator and weighed. The dried residue was subsequently incinerated in a muffle furnace (Milestone PYRO, Italy) at 550°C until a white ash was obtained. The sample was again cooled in a desiccator, and the final weight was recorded.

Crude fiber content was calculated using the formula:

Crude fiber%by weigh=W12−W×100W,

where *W*1 is weight of the Gooch crucible with contents before ashing (g), *W*2 is the weight of the Gooch crucible with celite and ash (g), and *W* is weight of the dried material before ashing (g).

#### 2.4.5. Determination of Fat Content

The total fat content of *Chakhao* rice samples was determined using Soxhlet extraction with petroleum ether as the solvent, following the standard gravimetric method described by Shin et al. [17]. The analysis was performed using a Gerhardt Soxtherm apparatus (SOX 6‐place, Germany), ensuring efficient and reproducible extraction of lipids. The procedure involved weighing an empty extraction thimble (*W*1), followed by the transfer of 10 g of the rice flour sample into the thimble. The weight of the thimble containing the sample was recorded (*W*2). The thimble was then placed inside a Soxhlet extraction unit (Gerhardt Soxtherm, SOX 6‐place, Germany), and sufficient petroleum ether was added to completely submerge the sample. The extraction was carried out until complete lipid extraction was achieved. After extraction, the remaining traces of petroleum ether were removed by evaporation in a drying oven (BTI‐HAO‐50, India) at 60–70°C for 30 min. The extraction flask was then cooled to room temperature and reweighed (*W*4).

The fat content (%) was calculated using the formula:

Fat%=weight of ether extract×100weight of the sample.

#### 2.4.6. Estimation of Carbohydrates

The total carbohydrate content of *Chakhao* rice samples was estimated using the difference method, a standard approach in proximate analysis. The percentage of carbohydrates was determined by subtracting the sum of the percentages of moisture, fat, protein, and total ash from 100%. This method assumes that the remaining fraction primarily consists of carbohydrates, including starch, sugars, and dietary fiber.

The carbohydrate content (%) was calculated using the formula:

Carbohydrate%=100−%moisture+%fat+%protein+%total ash.

### 2.5. Mineral Composition

The mineral composition of *Chakhao* rice samples was determined using ICP‐MS, performed on a Perkin Elmer Nexion 2000B (Serial No. 815N020403B). The system was coupled with a microwave digester (Titan 16 POS SYS, India) for sample digestion, and all sample weights were measured using a Precisa 205A SCS analytical balance (0.0001–250 g). Sample preparation and digestion were carried out following standard protocols [18].

For digestion, 0.2 g of the finely ground rice sample was accurately weighed into a digestion vessel. A mixture of 6 mL nitric acid (HNO_3_) and 3 mL Milli‐Q water was added to the vessel. The digestion vessel was covered and kept in a fume hood at room temperature for 10 min to allow pre‐reaction. The sample was then transferred to the microwave digestion system (Titan 16 POS SYS, India), where it underwent digestion at controlled conditions for 30–75 min, ensuring complete mineralization of organic material. After digestion, the vessel was cooled to near ambient temperature to reduce internal pressure. The digestion vessel was removed from the microwave system and placed in the fume hood for further cooling. Once brown fumes were no longer visible, the vessel was carefully opened, and Milli‐Q water was used to rinse the lid and inner walls, ensuring complete transfer of the digested sample. Using the standard curve of standard minerals as a guide, the quantity of each trace element was determined. The final solution was transferred into a centrifuge tube container for further analysis. The accuracy of the proposed method was evaluated by analyzing certified reference materials (CRMs) from the National Institute of Standards and Technology (NIST).

To assess potential contamination, a reagent blank containing the same acid mixture but without a sample was prepared alongside the test samples. The blank solution was analyzed to ensure the accuracy of mineral quantification.

### 2.6. Volatile Compounds

The fatty acid profiles and volatile flavor compounds in *Chakhao* rice samples were analyzed using gas chromatography‐tandem mass spectrometry (GC‐MS/MS) on a Shimadzu GCMS TQ8050 system. The separation was performed on an SH‐I‐5SIL MS column (30 m × 0.250 mm ID, film thickness 0.25 *μ*m) using helium as the carrier gas. A split injection (1 *μ*L injection volume) was utilized for sample introduction. The ion source temperature was set to 220°C, ensuring optimal ionization and compound identification through mass spectrometry.

FAMEs in the *Chakhao* samples were analyzed by following a standard methylation protocol [19]. The transesterification reagent was prepared by dissolving 12.5 g of potassium hydroxide (KOH) in 10 mL of distilled water, followed by the addition of 90 mL of methanol. For FAME preparation, 100 mg of the sample was accurately weighed and mixed with 4–5 mL of KOH‐methanol solution. The mixture was vortexed for 2 min to initiate the transesterification process. A 2 mL of hexane was then added, and the mixture was vortexed again to facilitate phase separation. The sample was centrifuged at 4°C for 5 min using a refrigerated centrifuge. Following centrifugation, the upper organic layer containing FAMEs was collected and used for GC‐MS/MS analysis.

FAMEs in *Chakhao* rice samples were analyzed using gas chromatography (GC) equipped with an Rtx‐2560 capillary column (ID 1022, 0.20 *μ*m film thickness). This analysis provides a standardized method for quantifying and comparing fatty acid profiles across different *Chakhao* samples. The quantification of FAMEs was performed using the Shimadzu standardized method, ensuring precision and reproducibility. The percentage of each FAME was calculated using the following equation:

FAME %=Area%∗factor∗%of fat100,

where area % represents the relative peak area obtained from the chromatogram. Factor is a correction coefficient based on standard FAME calibration. % Fat refers to the total lipid content in the sample.

The determination of FAMEs is essential for ensuring product quality, consistency, and regulatory compliance, particularly for the commercialization or export of *Chakhao* based products. Furthermore, understanding the fatty acid composition is critical for assessing oxidative stability, as lipid oxidation can adversely affect the sensory attributes, flavor quality, consumer acceptability, and shelf life of the rice [19]. The data generated from this analysis also contribute to optimizing processing and storage conditions to minimize oxidation and preserve product integrity.

Volatile flavor compounds were analyzed using headspace gas chromatography (HS‐GC) with a purge‐and‐trap method [20]. The sample was homogenized before analysis, and 2 mg of sample was accurately weighed into a sealed vial using a crimper. The sealed vial was placed in the automated sample tray of the headspace sampler, where it was temperature‐controlled to ensure uniform equilibrium of volatile compounds. After equilibration, the headspace gas was automatically injected into the GC‐MS/MS system for analysis. By matching mass spectra with the data in the NIST Mass Spectral Library, volatile compounds were identified.

### 2.7. Statistical Analysis

All experiments were conducted in triplicate, and the results were expressed as mean values ± standard deviation (SD). Data were statistically analyzed using SPSS software (Version 22.0, IBM); ANOVA (one way analysis of variance) was done to determine the significant differences between the mean values, and Tukey′s HSD post hoc method was employed to analyze the significant differences between the samples. *p* values < 0.05 were considered statistically significant.

## 3. Results and Discussion

### 3.1. Proximate Composition

The proximate composition of *Chakhao* rice samples from five different locations is presented in Table 1, detailing moisture, fat, protein, carbohydrates, total ash, acid‐insoluble ash, and crude fiber content.

**Table 1:** Proximate composition of *Chakhao* grown in five different regions in Manipur.

Samples	Moisture	Protein	Fat	Carbohydrate	Total ash	Crude fiber
Kakching	11.47 ± 0.21^c^	10.43 ± 0.22^b^	2.46 ± 0.48^b^	74.07 ± 0.89^a^	1.55 ± 0.05^a^	2.21 ± 0.01^b^
Chingarel	11.84 ± 0.12^b^	10.22 ± 0.10^c^	2.41 ± 0.05^b^	74.21 ± 0.39^a^	1.29 ± 0.07^b^	1.99 ± 0.00^c^
Tellou Chana	11.51 ± 0.30^b^	10.89 ± 0.10^a^	2.39 ± 0.45^c^	73.88 ± 0.76^b^	1.32 ± 0.11^b^	2.30 ± 0.09^a^
Khundrakpam	12.09 ± 0.11^a^	10.19 ± 0.17^c^	2.77 ± 0.11^a^	73.94 ± 0.39^b^	0.99 ± 0.00^c^	2.40 ± 0.07^a^
Chandel	11.31 ± 0.02^c^	10.41 ± 0.20^b^	2.32 ± 0.43^c^	74.26 ± 0.77^a^	1.69 ± 0.06^a^	2.08 ± 0.11^b^

*Note:* Mean ± SD; *n* = 3. Superscript letters indicate statistically significant differences (*p* < 0.05).

Moisture content ranged from 11.31% to 12.09%, which is within the safe storage threshold of ≤ 12% for rice grains. Moisture levels influence microbial activity, enzymatic reactions, and oxidative processes, affecting shelf life and grain quality. Exceeding 12% moisture can increase susceptibility to fungal contamination, mycotoxin formation, and insect infestation, whereas lower moisture levels can lead to mechanical damage during milling [21]. The observed differences in moisture content may be attributed to variations in postharvest drying techniques, storage conditions, and environmental humidity.

Protein content ranged from 10.19% to 10.89%, which is in line with previously reported values for *Chakhao*, 10.66% [22], and 11.06% [23]. Protein concentration in rice is influenced by genetic factors, soil nitrogen availability, and fertilization practices. Higher nitrogen uptake promotes amino acid biosynthesis and protein accumulation, whereas deficiencies can limit grain protein content. The minimal variability across samples suggests that agronomic conditions and genetic stability contribute to uniform protein levels in *Chakhao*. The protein content was analyzed as a proxy for nitrogen assimilation, though free and total amino acid profiles were not determined, which was a limiting factor. Future study containing amino acid quantification would provide more understanding of nitrogen′s role in grain quality.

Fat content ranged from 2.46% to 2.94%, consistent with earlier reports on pigmented rice but lower than brown rice, which contains 1.72%–3.37% fat [24]. Lipids in rice grains contribute to flavor development, oxidative stability, and nutritional quality. The fat content in *Chakhao* is primarily derived from the bran and germ, making it susceptible to oxidative rancidity. Similarly, a previous study from Indonesia reported fat content of 2.75%–2.85% for black rices from Jawa Islands [25]. Differences in fat content can result from milling intensity, exposure to oxygen, and environmental stress during grain development.

Crude fiber content ranged from 1.99% to 2.40%, reflecting contributions from bran layers, cell walls, and polysaccharides. Similarly, a previous study revealed 2.52% of crude fiber in black rice [26], and stated that the crude fiber content can vary depending on the milling condition. Dietary fiber plays a critical role in regulating digestion, modulating glycemic response, and enhancing gut microbiota. The high fiber content in *Chakhao* than other rice varieties suggests better retention of the outer grain layers, as fiber content typically decreases with increased milling and polishing [27].

Total ash content, which represents total mineral content, ranged from 0.99% to 1.69%, indicating differences in mineral uptake efficiency, soil composition, and agronomic practices. Similarly, Paiva et al. [28] reported 0.6% total ash content from the black rice of Brazil. The observed variability in ash content suggests region‐specific differences in soil nutrient availability. Higher ash content correlates with greater retention of essential minerals such as potassium (K), iron (Fe), magnesium (Mg), and calcium (Ca), which are crucial for metabolic processes and human nutrition.

Carbohydrate content ranged from 73.88% to 74.26%, which aligns with standard carbohydrate concentrations in pigmented rice varieties. Sompong et al. [29] observed carbohydrate content of 71.99%–75.71% (dry matter basis) for black rice, which is in line with our findings. Starch, the predominant carbohydrate component, determines cooking quality, gelatinization properties, and energy availability. The stability in carbohydrate content across samples indicates minimal environmental influence on starch biosynthesis, which is primarily controlled by genetic expression and enzymatic activity during grain filling.

The proximate analysis confirms that *Chakhao* rice exhibits consistent macronutrient composition, with variations in moisture, fat, and minerals, which can be influenced by the environmental conditions, processing methods, and postharvest handling [30]. These proximate variations are not only unique to *Chakhao* but also reported in different cereals [31].

### 3.2. Mineral Composition

The mineral composition of *Chakhao* rice samples from five different regions of Manipur is presented in Table 2. The study identified K, Mg, and Ca as the most abundant minerals, consistent with previous findings on black rice mineral composition [32]. Comparative studies have established that black rice generally contains higher concentrations of Fe, zinc (Zn), manganese (Mn), and phosphorus (P) than conventional white rice, reinforcing its superior nutritional profile [33].

**Table 2:** Mineral composition of *Chakhao* grown in five different regions of Manipur.

Minerals	Kakching	Chingarel	Telok Chana	Chandel	Khundrakpam
Na	0.63 ± 0.02^b^	0.43 ± 0.02^c^	1.03 ± 0.01^a^	0.41 ± 0.01^c^	0.70 ± 0.01^b^
Mg	135.06 ± 0.04^b^	121.55 ± 0.02^c^	130.26 ± 0.03^b^	93.88 ± 0.01^d^	145.74 ± 0.02^a^
K	300.74 ± 0.02^b^	291.16 ± 0.03^c^	308.10 ± 0.02^b^	238.14 ± 0.02^d^	348.42 ± 0.02^a^
Ca	14.54 ± 0.02^b^	9.75 ± 0.02^c^	15.19 ± 0.01^a^	11.76 ± 0.02^c^	13.79 ± 0.02^b^
Mn	2.72 ± 0.02^c^	2.85 ± 0.02^c^	4.43 ± 0.01^a^	2.21 ± 0.01^d^	3.75 ± 0.01^b^
Fe	6.63 ± 0.02^a^	1.11 ± 0.01*c*	6.50 ± 0.01^a^	0.80 ± 0.01^d^	0.93 ± 0.01^d^
Cu	0.14 ± 0.01^b^	ND	0.24 ± 0.01^a^	0.32 ± 0.01^a^	0.32 ± 0.01^a^
Zn	1.35 ± 0.01^c^	1.64 ± 0.02^a^	1.52 ± 0.01^b^	1.42 ± 0.01^b^	1.72 ± 0.02^a^
Se	ND	ND	0.02 ± 0.01^a^	0.02 ± 0.01^a^	ND

*Note:* Mean ± SD; *n* = 3. Different superscripts (^a^,^b^,^c^,^d^) indicate significant differences (*p* < 0.05). Same superscripts indicate no significant difference within that parameter. ND (not detected) values are excluded from statistical analysis. The values in the table is given in mg/kg.

K was detected in the highest concentrations, ranging from 238.14 to 348.42 mg/kg, which aligns with previously reported values for black rice (265.00–500.00 mg/kg) [30]. K is an essential macronutrient involved in cellular osmoregulation, enzyme activation, and carbohydrate metabolism. The observed variability in K levels across samples may be attributed to soil K availability, fertilization practices, and uptake efficiency by different cultivars.

Magnesium content ranged from 93.88 to 145.74 mg/kg, with Sample 5 exhibiting the highest concentration. Magnesium is crucial for enzyme activation, chlorophyll synthesis, and ATP metabolism in plants and plays a vital role in neuromuscular function and bone health in humans. Differences in magnesium concentration may result from soil composition and irrigation practices, as magnesium availability is influenced by soil pH and organic matter content.

Ca, another key mineral, exhibited significant variability across samples, with Sample 3 having the highest Ca content. Ca is essential for cell wall integrity, intracellular signaling, and structural development in rice grains. The variation in Ca levels may be linked to differences in soil Ca content and root absorption capacity.

Fe concentrations were significantly higher in Samples 1 and 3, whereas Sample 3 also had the highest Mn content. Mn is an essential cofactor for photosynthesis and enzyme activity, whereas Fe is critical for oxygen transport, hemoglobin synthesis, and enzymatic reactions. The observed variations in Fe and Mn levels across samples highlight the impact of altitude, soil composition, and organic matter interactions on micronutrient accumulation.

Selenium (Se) was detected at low concentrations across all samples, with Sample 2 exhibiting the highest Se content, whereas Sample 1 had the lowest. Se is an essential trace element involved in antioxidant defense and thyroid metabolism, though its uptake by plants is highly soil‐dependent. Copper (Cu) and Se were found in very low concentrations, with some samples showing undetectable levels, indicating that these elements are present in trace amounts in *Chakhao* rice.

The significant variability in mineral composition among samples suggests that altitude, soil nutrient availability, and agricultural practices influence the nutrient profile of *Chakhao*. Sample 3 exhibited the highest overall mineral content, whereas Sample 4 showed the lowest levels of Mg and K, reinforcing the role of environmental and edaphic factors in determining mineral accumulation in rice grains. The current study was limited to analyze the mineral profiling of *Chakhao* samples alone; future studies can analyze the mineral profiling of soil samples to compare the soil and grain as well as the bioaccumulation factors.

### 3.3. Flavor Profile

The volatile flavor profiles of *Chakhao* rice samples from five different regions of Manipur are presented in Table 3. The composition of volatile compounds in rice is influenced by genetic factors, environmental conditions, and postharvest processing methods, all of which contribute to its unique sensory attributes. A previous study on the volatile and sensory profiles of black rice identified 13 aldehydes, 13 ketones, 3 alcohols, 6 acids, esters, and an additional 16 volatile compounds [7].

**Table 3:** Common flavor compounds in the five different samples.

Compounds name	Kakching	Chingarel	Tellou Chana	Khundrakpam	Chandel
Hexanal	16.34	13.58	10.07	14.27	6.50
Nonanal	3.76	5.12	7.82	8.85	7.82
Butanal‐3‐methyl	4.04	4.31	5.57	6.02	1.41
Furan 2‐pentyl‐	4.22	4.31	2.95	3.92	2.05
1‐Hexanol	6.50	1.12	4.55	8.24	1.66
Azulene	ND	ND	12.52	14.36	ND

*Note:* The unit for all table values are represented in percentage (%).

Abbreviation: ND, not detected.

Aldehydes were among the most dominant volatile compounds detected in *Chakhao* samples. Hexanal, octanal, nonanal, and decanal were identified across all samples, with hexanal being particularly significant. Hexanal imparts a fresh, green, grassy aroma, commonly associated with rice [34]. Its consistent presence in all five samples suggests that it plays a fundamental role in defining *Chakhao*′s characteristic aroma. Octanal was also consistently detected, contributing to a strong citrus‐like scent with fresh and slightly waxy notes, whereas nonanal imparts a sweet, waxy aroma [7].

Acetic acid was found in all samples, contributing a pungent, vinegar‐like odor [7]. Acetic acid is typically formed through fermentation and lipid oxidation, and its presence in *Chakhao* suggests a role in enhancing the rice′s acidic and slightly sour undertones. The alcohols 1‐hexanol and 1‐octanol were also identified across all samples. 1‐hexanol contributes green, fruity, apple‐skin‐like aromas, whereas 1‐octanol is responsible for waxy, green, and citrus‐like notes. Their consistent detection across all samples suggests that these alcohols play a crucial role in shaping the overall flavor perception of *Chakhao*.

Monoterpenes, a class of highly volatile aromatic compounds, were detected in some samples. Limonene and linalool, two major monoterpene odorants reported in black rice, contribute citrusy and floral notes [35]. Limonene was detected in Samples 3, 4, and 5 at minor concentrations, whereas linalool was present only in Sample 2. The absence of these compounds in Sample 1 suggests potential variations in genotypic expression or environmental influences affecting monoterpene biosynthesis.

In addition to aldehydes and alcohols, the presence of furan‐2‐pentyl and various other aldehydes (butanal, heptanal, and pentanal) further contributed to the complex sensory profile of *Chakhao*. These compounds are associated with sweet, nutty, and slightly fruity aromas, enhancing the rice′s overall flavor depth. The variation in the retention area of detected compounds across different samples indicates natural differences in volatile composition, which may result from soil composition, climate variations, or postharvest storage conditions.

Even though all five samples belong to the same rice variety, differences in volatile compound concentrations suggest that regional factors, agronomic practices, and postharvest handling methods influence *Chakhao*′s final aroma.

### 3.4. FAME

The FAME composition of *Chakhao* rice samples from five different regions of Manipur is presented in Table 4. Fatty acid methyl esters play a crucial role in determining nutritional quality, oxidative stability, and flavor development in rice. The study identified oleic acid (C18:1n‐9), linoleic acid (C18:2n‐6), and palmitic acid (C16:0) as the predominant fatty acids across all samples, consistent with previous studies on black rice lipid composition.

**Table 4:** Common *FAME* profiles in the five different samples.

Compounds name	Kakching	Chingarel	Tellou Chana	Khundrakpam	Chandel
Tridecanoic acid	0.52	0.39	0.59	0.68	0.72
Palmitic acid	20.51	18.48	19.12	18.69	18.38
Stearic acid	2.25	3.00	3.12	2.42	2.18
9‐Octadecenoic acid	38.91	42.95	40.86	37.84	37.84
Nonadecanoic acid	0.81	1.08	0.51	1.00	0.46
11‐Octadecenoic acid	1.24	1.09	0.87	1.04	0.95
9,12,15‐Octadecatrienoic acid	1.87	1.46	1.76	2.23	2.55
1,8,11‐Heptadecatriene	32.49	30.27	31.64	34.59	35.48
9‐Hexadecenoic acid	0.28	ND	ND	ND	ND
Gondoic acid	0.45	0.56	ND	0.59	ND
Decanoic acid	ND	ND	ND	0.35	ND
9‐Octadecenoic acid	ND	ND	0.50	ND	0.54
7‐Hexadecenoic acid	ND	ND	ND	0.12	ND

*Note:* The unit for all table values are represented in percentage (%).

Abbreviation: ND, not detected.

Oleic acid was the most abundant monounsaturated fatty acid (MUFA) in all samples, with concentrations ranging from 37.00% to 44.04%. This is in line with earlier reports, where oleic acid content in rice grains ranged from 38.60% to 40.35% [36]. Oleic acid is known for its stability and role in reducing oxidative stress, which contributes to longer shelf life and potential health benefits. The observed variation in oleic acid levels may be attributed to differences in genetic factors, environmental conditions, and postharvest storage practices. Sample 2 exhibited the highest oleic acid content (42.41%), whereas Sample 5 had the lowest (37.40%), suggesting regional differences in lipid metabolism and fatty acid biosynthesis.

Linoleic acid was the predominant polyunsaturated fatty acid (PUFA) across all samples, with values ranging from 30.27% to 35.48%. Linoleic acid plays a vital role in cell membrane integrity and lipid metabolism but is highly susceptible to oxidation, which can influence the stability and sensory attributes of rice grains. The observed variation in linoleic acid content among samples indicates potential differences in fatty acid desaturation mechanisms, which are influenced by genetics and environmental factors [37]. Sample 5 contained the highest proportion of polyunsaturated fats (37.45%), whereas Sample 2 exhibited the lowest (31.86%).

Palmitic acid was the dominant saturated fatty acid (SFA) across all samples, with concentrations ranging from 18.38% to 20.51%, closely aligning with previously reported values (18.90% to 21.15%) [36]. Palmitic acid is essential for energy storage and membrane stability but is associated with higher oxidative susceptibility when compared with monounsaturated fats. The total saturated fat content was relatively stable across samples, ranging from 20.14% to 22.04%, suggesting consistent genetic regulation of saturated lipid biosynthesis.

The findings of this study align with earlier reports on the lipid composition of black rice, where triglycerides (TAGs) were found to be the predominant lipid class, composed of esterified oleic acid (42.10%), linoleic acid (29.30%), and palmitic acid (20.30%) [37]. The variability in monounsaturated and polyunsaturated fat concentrations across the samples suggests potential differences in enzymatic activity related to lipid metabolism, which may be influenced by temperature, altitude, and soil composition.

The fatty acid profiles observed in *Chakhao* suggest that it is a rich source of nutritionally beneficial unsaturated fats, particularly monounsaturated and PUFAs. The differences in lipid composition across samples highlight the influence of environmental factors on fatty acid biosynthesis.

## 4. Conclusion

This study provides a detailed characterization of *Chakhao* rice, emphasizing its nutritional and biochemical attributes. The proximate analysis revealed *Chakhao*′s high nutritional value. Mineral analysis showed that K, magnesium, and Ca were the most abundant minerals in *Chakhao*, with notable variability across samples due to geographical influences. Volatile analysis identified hexanal, nonanal, and octanal as the predominant aldehydes, contributing to its green, citrusy, and waxy aroma. Fatty acid profiling confirmed oleic acid as the dominant MUFA, followed by linoleic acid and palmitic acid, reinforcing its oxidative stability and health benefits. Though the amino acid and pigment profiling was a limitation, these findings establish *Chakhao* as a nutritionally rich and flavor‐distinctive rice variety, with significant potential for functional food applications and commercial valorization. The current study limits with the proximate, minerals, flavors, and FAMEs; further molecular analysis such as SSR markers in future studies provides the complementary information to compare and correlate with flavor related genes.

## Author Contributions

**Babli Waribam:** investigation, data curation. **Raju Sasikumar:** conceptualization, investigation, data curation, writing – original draft preparation, review, and editing. **Selva Kumar T.:** writing – original draft preparation. **Phani Kumar Garlapati:** software implementation, methodology development, and validation. **Sandeep Janghu:** project supervision. **S. Chakkaravarthi:** manuscript preparation. **Paul Mansingh:** experimentation and data interpretation. **Vidisha Tomer:** review and editing. **Amit K. Jaiswal:** conceptualization, validation, review, and editing.

## Funding

No funding was received for this manuscript.

## Ethics Statement

The authors have nothing to report.

## Consent

The authors have nothing to report.

## Conflicts of Interest

The authors declare no conflicts interest.

## Data Availability

The data that support the findings of this study are available from the corresponding authors upon reasonable request.
